# Oxidative Stress and Apoptosis in Disk Abalone (*Haliotis discus hannai*) Caused by Water Temperature and pH Changes

**DOI:** 10.3390/antiox12051003

**Published:** 2023-04-26

**Authors:** Min Ju Kim, Jin A Kim, Dae-Won Lee, Young-Su Park, Jun-Hwan Kim, Cheol Young Choi

**Affiliations:** 1 Department of Convergence Study on the Ocean Science and Technology, Korea Maritime and Ocean University, Busan 49112, Republic of Korea; ara0898@g.kmou.ac.kr (M.J.K.); jina@g.kmou.ac.kr (J.A.K.); 2 Marine Biotechnology and Bioresource Research Department, Korea Institute of Ocean Science and Technology, Busan 49111, Republic of Korea; dw0130@kiost.ac.kr; 3 Department of Nursing, Catholic University of Pusan, Busan 46252, Republic of Korea; yspark@cup.ac.kr; 4 Department of Aquatic Life and Medical Science, SunMoon University, Asan 31460, Republic of Korea; 5 Division of Marine BioScience, Korea Maritime and Ocean University, Busan 49112, Republic of Korea

**Keywords:** pH, water temperature, oxidative stress, apoptosis, shellfish

## Abstract

Ocean warming and acidification can induce oxidative stress in marine species, resulting in cellular damage and apoptosis. However, the effects of pH and water temperature conditions on oxidative stress and apoptosis in disk abalone are poorly understood. This study investigated, for the first time, the effects of different water temperatures (15, 20, and 25 °C) and pH levels (7.5 and 8.1) on oxidative stress and apoptosis in disk abalone by estimating levels of H_2_O_2_, malondialdehyde (MDA), dismutase (SOD), catalase (CAT), and the apoptosis-related gene caspase-3. We also visually confirmed apoptotic effects of different water temperatures and pH levels via in situ hybridization and terminal deoxynucleotidyl transferase dUTP nick end labeling assays. The levels of H_2_O_2_, MDA, SOD, CAT, and caspase-3 increased under low/high water temperature and/or low pH conditions. Expression of the genes was high under high temperature and low pH conditions. Additionally, the apoptotic rate was high under high temperatures and low pH conditions. These results indicate that changes in water temperature and pH conditions individually and in combination trigger oxidative stress in abalone, which can induce cell death. Specifically, high temperatures induce apoptosis by increasing the expression of the apoptosis-related gene caspase-3.

## 1. Introduction

The annual increase of approximately 2 ppm in global atmospheric CO_2_ levels is a consequence of the emission of 10 billion metric tons of carbon from the combustion of fossil fuels [1]. This has primarily contributed to a rise in seawater temperature, which is predicted to increase by up to +4 °C by 2100 [2]. The mid-latitude region where the Republic of Korea is located often experiences significant fluctuations in seawater temperature with the season. Along with global ocean warming, these fluctuations could have considerable adverse effects on nearby waters.

The marine environment serves as a major carbon sink, absorbing CO_2_ from the atmosphere into the ocean [3]. However, CO_2_ absorption triggers a reaction with water molecules, generating hydrogen cations (H^+^) [4]. As the concentration of H^+^ increases, the ocean pH decreases, leading to ocean acidification. Although the ocean pH generally ranges from 8.1 to 8.2, it is projected to drop to approximately 7.7 by 2100 [2].

Ocean acidification and warming are interconnected phenomena that occur simultaneously. Thus, it is crucial to investigate the complex effects of multiple environmental factors, such as pH and water temperature, on marine life.

Changes in water temperature and pH have negative impacts on various physiological aspects of marine organisms, including growth, survival, immune system, and food chain stability [5]. These environmental factors can negatively affect several physiological aspects, including the oxidative balance of marine organisms. Oxidative stress due to changes in these environmental factors triggers the production of reactive oxygen species (ROS), such as superoxide radicals (O_2_^−^), H_2_O_2_, and hydroxyl radicals (HO^−^), which oxidize several intracellular substances, including DNA [6]. ROS generation can cause lipid peroxidation (LPO) and produce malondialdehyde (MDA) (as a by-product) and damage the DNA [7,8]. This phenomenon also induces apoptosis through the caspase activation pathway [9]. Caspase-3 is the final gene in the apoptosis signal transduction mechanism that transmits the signal for apoptosis due to stress [10]. Ammonia stress can increase caspase-3 and trigger apoptosis in organisms [11]. Marine organisms have evolved and developed various mechanisms to counteract these negative effects [12]. Antioxidant enzymes, such as superoxide dismutase (SOD) and catalase (CAT), help remove toxic ROS. SOD converts O_2_^−^ into H_2_O_2_, which is then converted into stable H_2_O and O_2_ molecules by CAT [13]. Zhou et al. [14] reported that Mn stress caused oxidative stress in fish by detecting the levels of H_2_O_2_, CAT, and SOD. Additionally, Cui et al. [15] measured SOD, CAT, MDA, and ROS and found that the harmful substance 4-tert-butylphenol induced oxidative stress in common carp (*Cyprinus carpio* L.).

Recent studies have analyzed the combined effects of global warming and ocean acidification on various marine organisms [16,17] and found that their combined effects exert stronger impacts on the physiology of shellfish than global warming alone [18,19]. Therefore, it is crucial to investigate the physiological responses of marine organisms to the combined effects of these factors to comprehend their impacts. Abalone (*Haliotis discus hannai*), a valuable shellfish in both domestic and international markets, is found in coastal areas that experience substantial environmental changes [20], making it an appropriate species for experimental research. While previous studies have examined the effects of changes in the marine environment on shellfish, they have primarily focused on survival rate, oxygen consumption, and energy metabolism. Additionally, the majority of studies that have investigated the impacts of global warming and ocean acidification have been conducted on bivalves, such as mussels and scallops [21,22], with most gastropod studies targeting Haliotis species [23,24,25]. To the best of our knowledge, there are no studies investigating the effects of changes in water temperature and pH on gastropods. Furthermore, few studies have explored the mechanisms underlying oxidative stress-induced changes in the physiology of abalones exposed to various marine environmental changes.

In this study, we aimed to investigate the impact of different pH and water temperature conditions on the antioxidant response and apoptosis in *H. discus hannai*. We measured the levels of oxidative stress biomarkers (H_2_O_2_ and MDA) in the hemolymph and the expressions of antioxidant enzymes (SOD and CAT) in the hepatopancreas of abalone under various conditions. Additionally, we determined the degree of apoptosis by measuring caspase-3 expression and confirmed the results visually through in situ hybridization and terminal deoxynucleotidyl transferase dUTP nick end labeling (TUNEL) assays.

## 2. Materials and Methods

### 2.1. Experimental Organism

*H. discus hannai* (body length: 7.96 ± 0.52 cm, mass: 50.07 ± 6.14 g) were purchased from an abalone aquafarm (Naegohyang nongsusan, Wando, Republic of Korea), transferred to the laboratory, and acclimated for 1 week in six 350-L recirculating filtration tank systems (12 abalones per tank). Sufficient feed (dried kelp) was provided to the abalones twice a day during the acclimation period. Feeding was stopped 24 h prior to the experiment, which lasted 5 days. During the adaptation period, the seawater was maintained at a salinity of 34 ± 0.7 psu, a temperature of 20.2 ± 1.2 °C, and a pH of 8.03 ± 0.8, with constant aeration.

### 2.2. Experimental Design

Each tank was subjected to different temperature and pH conditions (15, 20, and 25 °C and pH 8.1 and 7.5, alone and in combination), including a control group (pH 8.1 and 20 °C), low temperature (LT, pH 8.1 and 15 °C), high temperature (HT, pH 8.1 and 25 °C), ocean acidification (OA, pH 7.5 and 20 °C), ocean acidification combined with low temperature (OA+LT, pH 7.5 and 15 °C), and ocean acidification combined with high temperature (OA+HT, pH 7.5 and 25 °C). Please refer to Figure S1 for a schematic of the experimental water tanks used in this study. The pH of the seawater was adjusted using a pH controller (Tunze Smart Controller 7000; Aquarientechnik GmbH, Penzberg, Bavaria, Germany), and a pH sensor was installed in the sump tank connected to the pH controller to achieve a pH of 7.5. A ceramic CO_2_ diffuser was connected to the CO_2_ cylinder via a high-pressure hose. Half of the total seawater was replaced daily with fresh seawater adjusted to the appropriate pH level for the experiment to maintain a constant pH. The seawater’s pH level, temperature, and atmospheric pCO_2_ were measured daily using a pH controller (Pro2030; YSI Inc., Yellow Springs, OH, USA) and a pump-aspirated sampling-type CO_2_ meter (GM70; Vaisala, Vantaa, Finland), respectively. The seawater physio-chemical parameters were calculated using CO_2_SYS with the constants of Dickson and Millero [26], as described in Table S1.

### 2.3. Sampling

Three abalones were selected at random from each experimental tank. Abalones were collected randomly from each triplicate experiment on days 1, 3, and 5 of exposure. The abalones in each sample were anesthetized using MS-222 (ethyl 3-aminobenzoate methanesulfonate salt, A5040; Sigma, Saint Louis, MO, USA). The hemolymph was immediately collected from the pallial sinus using a 1 mL sterile syringe (26G; Jungrim, Seoul, Republic of Korea) and then promptly centrifuged at 4 °C and 12,000× *g* for 10 min. The supernatant was used to analyze the hemolymph parameters. Hepatopancreatic tissue samples collected from the abalones on days 0, 1, 3, and 5 were used for total RNA extraction and fixed at 20 °C in 10% formalin and 4% paraformaldehyde (PFA) prior to TUNEL assay and in situ hybridization, respectively.

### 2.4. Analysis of Hemolymph Parameters

To quantify the amount of ROS generated in the abalones, a commercial kit (PeroxiDetect™ Kit, PD1-1KT; Sigma, St. Louis, MO, USA) was used to detect the H_2_O_2_ level. The level of MDA was measured by following the manufacturer’s protocol for the OxiTec™ TBARS (LPO) Assay Kit (BO-TBR-200; BIOMAX, Guri-si, Gyeonggi-do, Republic of Korea). Finally, the concentrations of H_2_O_2_ and MDA in each sample were determined by measuring the sample’s absorbance at 520 nm using the 2030 multilabel reader (Victor X3, 2103-0010; Perkin Elmer, Waltham, MA, USA).

### 2.5. Total RNA Extraction and Complementary DNA Synthesis

Total RNA was extracted from the hepatopancreatic tissue of the disk abalone on days 0, 1, 3, and 5 using TRI Reagent^®^ (TR188, Molecular Research Center; Cincinnati, OH, USA), following the manufacturer’s instructions. The RNA purity was calculated to be approximately 1.9−2.0 (A_260_/A_280_), and the degree of contamination was approximately 2.0−2.2 (A_260_/A_230_). Next, 2 μg of total RNA was reverse transcribed into complementary DNA (cDNA) using an oligo-(dT)_15_ anchor primer (Cosmogenetech; Seoul, Republic of Korea) and the Moloney Murine Leukemia Virus (M-MLV) cDNA Synthesis Kit (EZ006S; Enzynomics, Daejeon, Republic of Korea), according to the manufacturer’s instructions. All synthesized cDNA samples were stored at −20 °C and diluted with distilled water at a 1:50 ratio before conducting real-time polymerase chain reaction analysis.

### 2.6. Real-Time Quantitative Polymerase Chain Reaction

To measure the relative expression of SOD, CAT, caspase-3, and β-actin mRNA in the hepatopancreas, real-time polymerase chain reaction (qPCR) was conducted using the Bio-Rad iCycler iQ multicolor real-time PCR detection system (Bio-Rad, Hercules, CA, USA) and iQ SYBR Green Supermix (Bio-Rad) as per the manufacturer’s guidelines. The mRNA sequences available from the National Center for Biotechnology Information (NCBI) were utilized to design the primers for qPCR (Table S2). β-actin was used as an internal control to compare the relative mRNA expression levels with other genes. qPCR was performed with 0.5 μL of cDNA, 0.25 μM of each forward and reverse primer, 0.2 mM of dNTP, 10 μL of SYBR Green, and Taq polymerase with 10× reaction buffer (10 mM of Tris-HCl at pH 9.0, 50 mM of KCl, 1.4 mM of MgCl_2_, and 20 nM of fluorescein) in the 25-μL reaction solution. The thermal cycling process was set at one cycle of denaturation at 95 °C for 5 min, 30 cycles of denaturation at 95 °C for 20 s, and annealing at 53.4 °C for 20 s. The data were presented as changes relative to the cycle threshold (ΔCt) levels calculated with respect to β-actin. The 2^−ΔΔCt^ method [27] was used to calculate the calibrated ΔCt (cycle threshold) value (ΔΔCt) for each sample and internal control (β-actin mRNA expression data of each tissue).

### 2.7. In Situ Hybridization Detection of SOD mRNA

The SOD sequence used for the in situ hybridization probe was designed (Table S2) and amplified via PCR before being inserted into the pGEM-T Easy Vector (A137A; Promega, Madison, WI, USA). After confirming the antisense sequence via sequencing, the plasmid DNA was amplified using PCR with the antisense, sense, and T7 primers (5ʹ-TAA TAC GAC TCA CTA TAG GG-3ʹ). DIG-labeled RNA probes were generated using a DIG RNA labeling mix (Merck, Darmstadt, Germany), with the PCR products obtained from the antisense primer and T7 RNA polymerase (Merck, Darmstadt, Germany) being used as the antisense labeling probes.

For the preparation of frozen section tissue slides, hepatopancreatic tissues of the disk abalones from all experimental groups were collected on day 5 (pH 8.1 and 15 °C, pH 8.1 and 20 °C (control), pH 8.1 and 25 °C, pH 7.5 and 15 °C, pH 7.5 and 20 °C, and pH 7.5 and 25 °C) and fixed in 4% paraformaldehyde (PFA) overnight at 4 °C. Following fixation, the samples were washed with PBS and stored in 30% sucrose to prevent ice-crystal formation. Next, the sections were subjected to hybridization with a hybridization buffer consisting of deionized formamide, 20× saline sodium citrate, 0.1% Tween-20, 1 M citric acid, and yeast tRNA (1:100 with hybridization buffer), along with the RNA probe, for a duration of 18 h at 65 °C.

For detection of the hybridization signal, tissue sections were initially incubated with a blocking solution containing calf serum in PBST for 1 h at 20 °C. Subsequently, the sections were incubated at 4 °C with an anti-digoxigenin antibody conjugated with alkaline phosphatase (1:2000 in blocking solution; Roche, Basel, Switzerland). After a series of washing steps in PBST, the sections were rinsed in alkaline-Tris buffer (1 M Tris at pH 9.5, 1 M MgCl_2_, 5 M NaCl, and 10% Tween-20), and color was developed using a labeling mix (alkaline Tris buffer, nitro-blue tetrazolium, and 5-bromo-4-chloro-3-indolyl phosphate disodium salt). Finally, the sections were washed with PBST, mounted with Aqua Polymount (Polysciences, Warrington, PA, USA), and observed under a stereomicroscope (Nikon Eclipse Ci, Tokyo, Japan) for image capture.

### 2.8. Terminal Deoxynucleotidyl Transferase dUTP Nick End Labeling (TUNEL) Assay

Hepatopancreatic tissues of the disk abalones from the experimental groups with pH 8.1 and 20 °C (control), pH 8.1 and 15 °C, pH 8.1 and 25 °C, pH 7.5 and 15 °C, pH 7.5 and 20 °C, and pH 7.5 and 25 °C were collected on day 5 to compare the effects of high/low temperature and low pH at the longest exposure duration. Meanwhile, hepatopancreatic tissues of the disk abalones from the pH 7.5 and 15 °C, pH 7.5 and 20 °C and pH 7.5 and 25 °C groups were also collected on days 0 (0-time control), 3, and 5 to compare the effects of high/low temperature and exposure time at low pH conditions. The hepatopancreatic tissues were fixed in 10% formalin at 20 °C. The samples were embedded in paraffin blocks after fixation. A tissue slide was prepared by sectioning the paraffin blocks. TUNEL staining was performed using a TUNEL assay kit (DeadEnd Colorimetric TUNEL System, G7131; Promega, Madison, WI, USA). The area of apoptotic signals was observed under an upright microscope to detect the degree of apoptosis (Nikon Eclipse Ci; Tokyo, Japan). The area was measured using the Image J program (LOCI, University of Wisconsin, Madison, WI, USA), and the average values were calculated.

### 2.9. Statistical Analysis

The difference between the analyzed data was determined using SPSS version 25.0 (IMB SPSS Inc., Chicago, IL, USA). Three-way analysis of variance (ANOVA) followed by Tukey’s post-hoc test was used for all data analyses to determine the interaction effects of temperature, pH, and exposure time (Table S3). Significant differences in the analyzed parameters were considered at *p* < 0.05. Values are expressed as the mean ± standard error (SE).

## 3. Results

### 3.1. Survival of Abalones

The different pH and temperature conditions did not affect the survival of abalones throughout the experiment (days 0, 1, 3, and 5).

### 3.2. Changes in H_2_O_2_ and MDA Levels in Hemolymph

**Figure 1:**
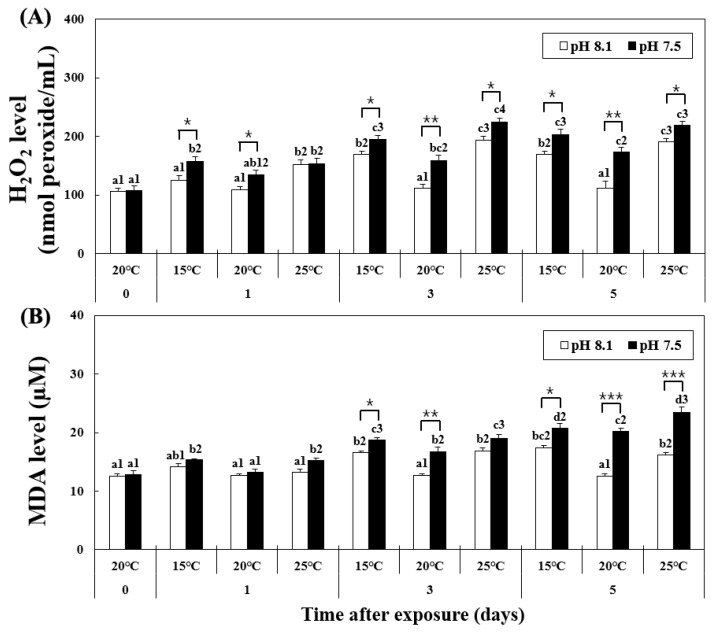
Changes in (**A**) MDA and (**B**) H_2_O_2_ levels in the hemolymph of *H. discus hannai* exposed to different pH and temperature conditions for 5 days. Different letters indicate significant differences among abalone groups exposed to the same temperature and pH for different times (*p* < 0.05). Numbers indicate significant differences under different temperatures for the same exposure time and pH (*p* < 0.05). The symbol “*” indicates a significant difference in MDA or H_2_O_2_ level between pH levels at the same temperature and time point (*, *p* < 0.05; **, *p* < 0.01, and ***, *p* < 0.001). All values represent the mean ± standard error (*n* = 3).

The level of H_2_O_2_ increased in the HT, OA, OA+LT, and OA+HT groups from day 1, as shown in Figure 1. At pH 8.1, the H_2_O_2_ level significantly increased from day 1 in the HT group and remained elevated on days 3 and 5 (pH 8.1 and 25 °C group on day 1 H_2_O_2_: 152.2 ± 7.03, day 3: 194.01 ± 6.39, day 5: 190.30 ± 5.60 nmol peroxide/mL, *p* < 0.05). Conversely, in the OA group, the H_2_O_2_ level increased from day 3 (pH 7.5 and 20 °C group on day 0: 133.88 ± 8.10 and on day 3: 159.09 ± 9.02 nmol peroxide/mL, *p* < 0.05). The H_2_O_2_ level also increased in the OA+LT and OA+HT groups from day 1 (pH 7.5 and 15 °C group: 156.80 ± 8.21, pH 7.5 and 25 °C group: 153.70 ± 9.09 nmol peroxide/mL on day 1, *p* < 0.05). Significant differences in H_2_O_2_ levels were observed between all groups with the same temperature and duration of exposure (e.g., LT group and OA+LT group on day 1) with changes in pH between each group, except for the control-OA group on day 0 and HT-OA+HT groups on day 1 (*p* < 0.05, *p* < 0.01, and *p* < 0.001).

At pH 8.1, the MDA level increased from day 3 to day 5 in the LT and HT groups (Figure 1). However, at pH 7.5, the MDA level continued to increase in the OA+LT and OA+HT groups from day 1 (pH 7.5 and 15 °C group: 15.32 ± 0.17, pH 7.5 and 25 °C group: 15.24 ± 0.43 μM on day 1, *p* < 0.05). In particular, the MDA levels were higher in the OA+HT group than in the OA+LT group on day 5 (pH 7.5 and 15 °C group: 20.76 ± 0.77, pH 7.5 and 25 °C group: 23.49 ± 0.86 μM on day 5, *p* < 0.05). The MDA level increased in the OA group from day 3 (pH 7.5 and 20 °C group on day 0: 13.21 ± 0.58, day 3: 16.78 ± 0.73 μM, *p* < 0.05). MDA levels differed significantly between all groups with the same temperature and duration of exposure (e.g., LT group and OA+LT group on day 3) with changes in pH between each group, except for the control-OA group on day 0 and all experimental groups on day 1, and HT-OA+HT groups on day 3 (*p* < 0.05, *p* < 0.01, and *p* < 0.001).

### 3.3. mRNA Expression of Antioxidant Enzymes

**Figure 2:**
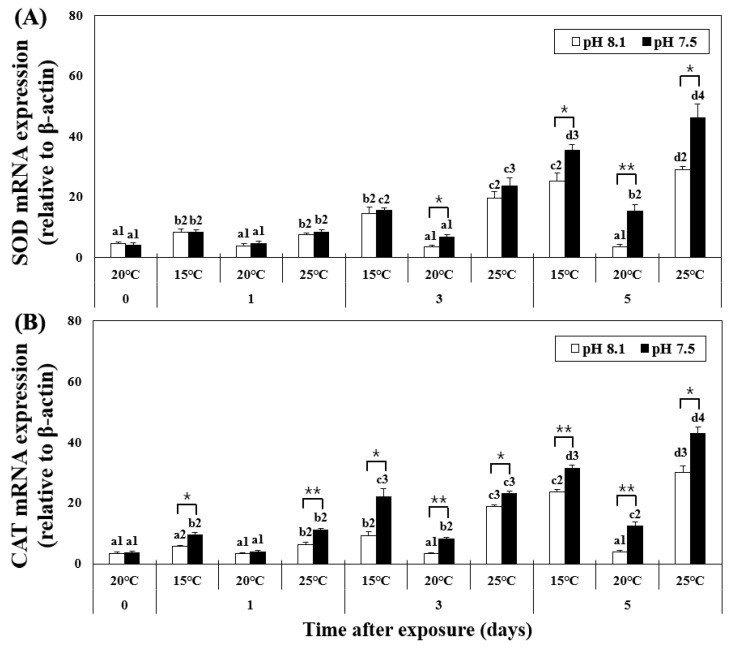
Changes in mRNA expression of (**A**) SOD and (**B**) CAT in the hepatopancreas of *H. discus hannai* exposed to different pH and temperature conditions for 5 days. Different letters indicate significant differences among abalone groups exposed to the same temperature and pH for different times (*p* < 0.05). Numbers indicate significant differences among different temperatures for the same exposure time and pH (*p* < 0.05). The symbol “*” indicates a significant difference in SOD or CAT mRNA expression between pH levels at the same temperature and time point (*, *p* < 0.05; **, *p* < 0.01). All values represent the mean ± standard error (*n* = 3).

The mRNA expression of SOD in the hepatopancreas was analyzed using qPCR and showed increased levels from day 1 in the LT and HT groups (Figure 2), for OA+LT and OA+HT groups (pH 7.5 and 15 °C group: 8.35 ± 0.98, pH 7.5 and 25 °C group: 8.51 ± 0.65 on day 1, *p* < 0.05). On day 5, the expression further increased in the OA+HT group compared to that in the OA+LT group (e.g., pH 7.5 and 15 °C group: 35.57 ± 1.84, pH 7.5 and 25 °C group: 46.35 ± 4.46 on day 5, *p* < 0.05). In the OA group, the mRNA expression of SOD increased only on day 5 (day 0: 4.77 ± 0.74, day 5: 15.28 ± 2.15, *p* < 0.05). A significant difference in the mRNA expression of SOD was observed with pH changes between the groups on days 3 and 5. In addition, a significant difference in the mRNA expression of SOD was observed based on pH changes in the OA+HT and OA+LT groups on day 5 (*p* < 0.05, *p* < 0.01, and *p* < 0.001).

Similar to the trends of the H_2_O_2_ level, the mRNA expression of CAT increased in the LT group on day 3; however, in the HT group, it increased on day 1 and steadily increased until day 5. At pH 7.5, the expression levels of CAT continuously increased in the OA+HT and OA+LT groups from day 1 to day 5 (pH 7.5 and 15 °C group: 9.42 ± 0.91, pH 7.5 and 25 °C group: 11.26 ± 0.54 on day 1, *p* < 0.05). Similar to SOD, the mRNA expression of CAT was higher in the OA+HT group than in the OA+LT group on day 5 (pH 7.5 and 15 °C group: 31.57 ± 0.93, pH 7.5 and 25 °C group: 43.02 ± 2.00 on day 5, *p* < 0.05). In the OA group, the expression of CAT increased only on day 3 (day 0: 3.96 ± 0.51, day 3: 8.31 ± 0.50, *p* < 0.05). A significant difference was observed between all groups with the same temperature and duration of exposure (e.g., LT group and OA+LT group on day 1) based on changes in pH, except for the control-OA groups on day 1 (*p* < 0.05, *p* < 0.01, and *p* < 0.001).

### 3.4. Expression of SOD mRNA in the Hepatopancreas

**Figure 3:**
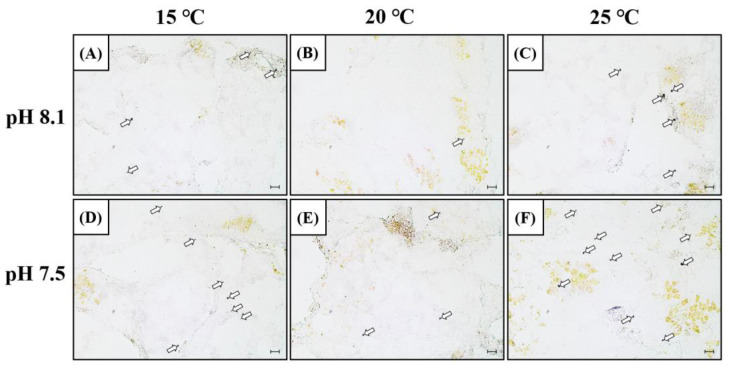
SOD mRNA expression in the hepatopancreas of *H. discus hannai* detected by in situ hybridization on day 5 (**A**–**F**). (**A**) pH 8.1 and 15 °C group; (**B**) pH 8.1 and 20 °C group; (**C**) pH 8.1 and 25 °C group; (**D**) pH 7.5 and 15 °C group; (**E**) pH 7.5 and 20 °C group; and (**F**) pH 7.5 and 25 °C group. Dark areas (white arrow) indicate the mRNA expression of SOD in the hepatopancreas. Scale bar = 25 μm.

In order to confirm the mRNA expression of SOD in the hepatopancreas, in situ, hybridization was performed using a SOD mRNA probe (Figure 3). The SOD mRNA signal intensity was detected in the cytoplasm of the hepatopancreatic tissue, and similar to SOD mRNA expression, it increased in the OA+LT and OA+HT groups on day 5. SOD mRNA signals were also observed in the LT, HT, and OA groups. Overall, the SOD mRNA signals were stronger in the OA+HT group than in the OA+LT group.

### 3.5. mRNA Expression of Caspase-3

**Figure 4:**
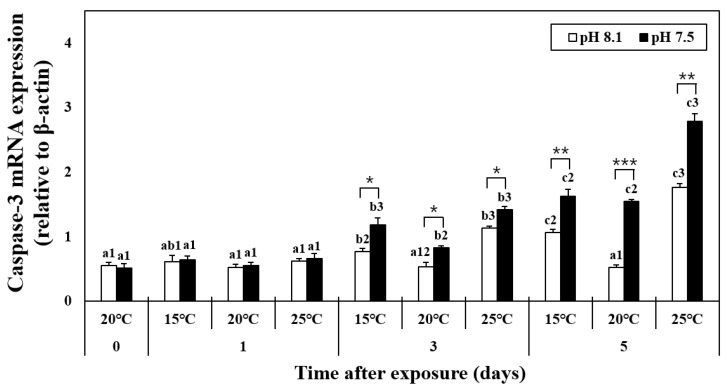
Changes in the mRNA expression of caspase-3 in the hepatopancreas of *H. discus hannai* exposed to different pH and temperature conditions for 5 days. Different letters indicate significant differences among abalone groups exposed to the same temperature and pH for different times (*p* < 0.05). Numbers indicate significant differences among different temperatures for the same exposure time and pH level (*p* < 0.05). The symbol “*” indicates a significant difference in caspase-3 mRNA expression between pH levels at the same temperature and time point (*, *p* < 0.05; **, *p* < 0.01, and ***, *p* < 0.001). All values represent the mean ± standard error (*n* = 3).

The mRNA expression of caspase-3 did not change on day 1 (Figure 4). In the HT, LT, OA, OA+HT, and OA+LT groups, the expression levels of caspase-3 increased from day 3 until day 5. In addition, it considerably increased in the OA+HT groups compared with the OA+LT groups on day 5 (pH 7.5 and 15 °C group: 1.63 ± 0.10, pH 7.5 and 25 °C group: 2.79 ± 0.12, day 5, *p* < 0.05). In the OA group, the mRNA expression of caspase-3 increased on day 3 (day 0: 0.56 ± 0.04, day 3: 0.82 ± 0.03, *p* < 0.05). A significant difference in caspase-3 expression levels was observed with the change in pH between the groups on days 3 and 5. A significant difference was also observed in all experimental groups with the change in pH, barring the control-OA group on day 0 and all temperature groups on day 1 (*p* < 0.05, *p* < 0.01, and *p* < 0.001).

### 3.6. Apoptosis Level in the Hepatopancreas

**Figure 5:**
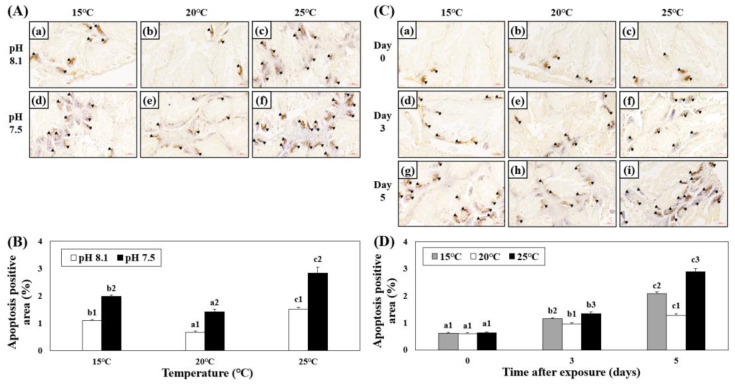
Images from the TUNEL assay of the hepatopancreatic tissue of *H. discus hannai* exposed to different pH and temperature conditions for the same exposure time (**A**,**C**). (**A**): (**a**) pH 8.1 and 15 °C; (**b**) pH 8.1 and 20 °C (control); (**c**) pH 8.1 and 25 °C; (**d**) pH 7.5 and 15 °C; (**e**) pH 7.5 and 20 °C; and (**f**) pH 7.5 and 25 °C on day 5. (**C**): [(**a**) pH 7.5, 15 °C; (**b**) pH 7.5, 20 °C; (**c**) pH 7.5, 25 °C on day 0]; ((**d**) pH 7.5, 15 °C; (**e**) pH 7.5, 20 °C; (**f**) pH 7.5, 25 °C on day 3); ((**g**) pH 7.5, 15 °C; (**h**) pH 7.5, 20 °C; and (**i**) pH 7.5, 25 °C on day 5). Black arrows (brown cells) indicate the apoptotic area. Scale bars = 60 μm. (**B**) Degree of apoptosis-positive area (%) in (**A**), and (**D**) Degree of apoptosis-positive area (%) in (**C**). Different characteristics in (**B**) indicate values corresponding to different temperatures within the same pH level, and the numbers in (**B**) indicate significant differences among different pH values at the same temperature (*p* < 0.05). Different letters in (**D**) indicate values corresponding to different exposure times for the same temperature, and numbers in (**D**) indicate significant differences among different temperatures within the same exposure times (*p* < 0.05). All values are presented as the mean ± standard error (*n* = 3).

The apoptotic areas in the hepatopancreatic tissues of abalones exposed to various combinations of temperature and pH were investigated using TUNEL analysis. The apoptotic area increased in the HT, LT, OA, OA+HT, and OA+LT groups on day 5. Among the experimental groups under acidic conditions, the apoptotic area was greater in the OA+HT group than that in the OA and OA+LT groups (Figure 5A,B). In addition, the apoptotic area in the hepatopancreas increased over time (Figure 5C,D), and it was the highest in the OA+HT groups than in other groups (pH 7.5 and 25 °C group on day 0: 0.63 ± 0.05, pH 7.5 and 25 °C group on day 5: 2.89 ± 0.10, *p* < 0.05).

## 4. Discussion

This study confirmed that changes in water temperature and pH-induced physiological stress responses, namely, increased ROS and MDA levels, in abalone. A previous study on Antarctic scallops (*Adamussium colbecki*) found that the complex environmental conditions of pH 7.6 (lower pH than the control) and 1 °C (water temperature higher than that of the habitat) increase ROS production and MDA level [28]. In both studies, the ROS and MDA levels increased under low pH and low/high water temperature conditions, indicating the occurrence of LPO and oxidative stress in abalone.

The mRNA expression levels of SOD and CAT increased under low pH and low/high water temperature conditions. In addition, the signal of SOD mRNA confirmed via in situ hybridization was consistent with the expression of SOD mRNA. Hu et al. [29] reported that SOD and CAT activities increase upon exposure of thick-shell mussels (*Mytilus coruscus*) to low pH (7.7 and 7.3) and high temperature (30 °C) environments. Furthermore, SOD activity increases in black tiger shrimp (*Penaeus monodon*) upon exposure to low (15 °C) or high (30 °C) water temperatures and this increase is more pronounced in high-temperature conditions [30]. Song and Choi [31] reported that the signal of SOD mRNA can be observed in the digestive gland of bay scallops (*Aregopcten irradians*) when exposed to a salinity-changing environment, which may cause oxidative stress. The results of these studies are consistent with the activities of antioxidant enzymes identified in the present study. Thus, pH and low/high water temperatures possibly act as environmental factors that trigger oxidative stress and increase ROS production. In addition, ROS production may be greater under the pH-complex environment conditions in high- than in the low-temperature environment. The activities of SOD and CAT possibly stabilized the increased ROS production in the abalones.

High expression levels of caspase-3, which transmits apoptotic signals and causes cell death, were observed with combined low pH and high temperature, similar to real global warming and acidification conditions. Our findings corroborate with that of Ong et al. [32] and Araújo et al. [33], which indicated that the two environments exerted negative synergistic effects, causing cell toxicity and increasing apoptosis. Wang et al. [34] reported that acidification increased caspase-3 gene activity in Pacific oyster (*Crassostrea gigas*) plasma, and Nash and Rahman [35] found that high-temperature conditions (32 °C) increased caspase-3 gene expression and apoptosis in American oyster (*Crassostrea virginica*). In addition, Zhang et al. [36] reported that cell death occurs in the top shell (*Trochus niloticus*) under low pH and high-temperature conditions (30 °C). These findings are similar to the results of the present study indicating that low pH and low/high water temperature conditions increased the caspase-3 mRNA expression and promoted apoptosis in disk abalone. As observed in previous studies, changes in environmental factors, such as pH and water temperature, trigger oxidative stress, ultimately inducing apoptosis by transmitting apoptotic signals through caspase-3.

Thus, for the first time, the present study focused on the effects of changes in water temperature and pH on disk abalone. The findings from this study suggest that oxidative stress may be greater when both environmental factors are altered simultaneously rather than separately. Oxidative stress, antioxidant activity, and apoptosis were generally elevated in abalone when both environmental factors were changed. These results indicated the negative synergistic effects of water temperature and pH on abalone, leading to apoptosis.

Another study reported that the effect of water temperature is greater than that of pH [22,37]. In the present study, changes in water temperature had a more rapid effect on abalone compared with changes in pH. In a low-pH environment, the stress response and antioxidant activity were higher in the abalones exposed to high temperatures than in those exposed to low temperatures. Oxidative stress and cell death were also greater under high temperatures than under low temperatures. These results indicate that high temperature is a greater stress factor than low temperature in abalone and can be attributed to the fact that abalones are sensitive to changes in water temperature.

## 5. Conclusions

In conclusion, our findings indicate the following: (1) water environment warming (high temperature) alone and in combination with acidification (low pH) promote oxidative stress in abalone; (2) considering the increase in ROS production due to oxidative stress under high-temperature conditions, the effects of acidification (low pH) combined with warming (high temperature) did not exceed those of warming alone; and (3) high temperature (25 °C) increased the levels of ROS and MDA as well as activities of antioxidant enzymes (SOD and CAT) and caspase-3 in abalone, thereby inducing apoptosis in the hepatopancreas.

As presented in this study, oxidative stress due to warming and acidification induces an antioxidant response and triggers apoptosis. This phenomenon disrupts the physiological balance of the body and can cause diseases and death of shellfish. Therefore, considering the response and adaptation of marine organisms to global climate change, further studies should investigate the impact of different environmental change scenarios on abalone based on the predictions of marine environmental changes. Such studies may contribute to predicting the physiological responses to changes in the aquatic environment and the vulnerability of shellfish according to changes in pH and water temperature, which do not migrate easily between water zones compared to fish.

## Data Availability

All relevant data are available within the manuscript.

## Supplementary Materials

The following supporting information can be downloaded at: https://www.mdpi.com/article/10.3390/antiox12051003/s1; Figure S1: Schematic of the experimental water tank; Table S1: Carbonate chemistry at each seawater condition; Table S2: Primers used for qPCR amplification; Table S3: Three-way ANOVA summary of the effects of temperature, pH and time on each analysis parameter.
