# Supplementary material for: Oxidative Stress and Apoptosis in Disk Abalone (Haliotis discus hannai) Caused by Water Temperature and pH Changes
Source: Antioxidants (Basel). 2023 Apr 26;12(5):1003. doi: 10.3390/antiox12051003 (PMC10215517; doi:10.3390/antiox12051003)
Supplement: Supplementary file 1 [file antioxidants-12-01003-s001.zip › antioxidants-2330294-supplementary.pdf]

Supplementary material - Figure S1. Schematic of the experimental water tank.

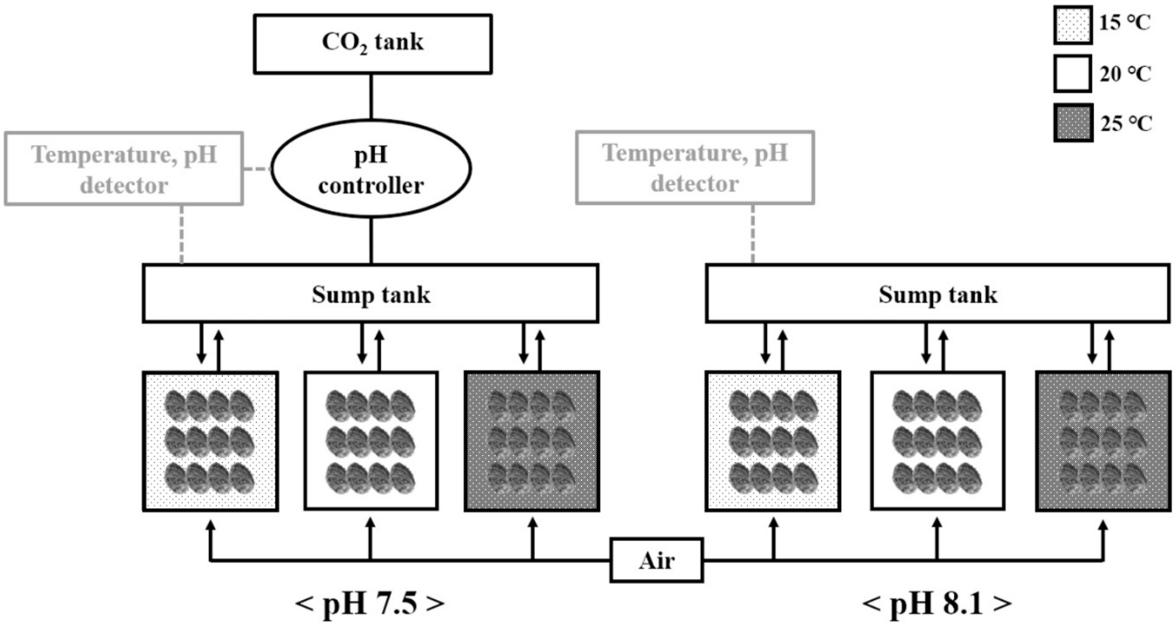

Supplementary material –

Table 1. Carbonate chemistry at each seawater condition. Salinity, temperature, pH_{NBS}, and pCO_2 were measured directly using equipment, and total alkalinity (A_T) was calculated by CO_2SYS (Lewis and Wallace, 1998). All values are presented as the mean ± standard error.

Desired condition		Salinity (psu)	Temperature (°C)	pH_{NBS}	pCO_2 (µatm)	A_T (mmol/kg)
pH 8.1	15 °C	34.53 ± 0.12	15.20 ± 0.08	8.09 ± 0.01	463.33 ± 2.72	2162.31 ± 66.58
	20 °C	34.33 ± 0.07	20.17 ± 0.10	8.09 ± 0.02	470.00 ± 8.16	2135.93 ± 62.76
	25 °C	34.53 ± 0.07	24.93 ± 0.07	8.09 ±0.01	466.67 ± 9.81	2067.78 ± 26.58
pH 7.5	15 °C	34.57 ± 0.11	15.20 ± 0.05	7.53 ± 0.01	1363.33 ± 24.19	1597.53 ± 77.59
	20 °C	34.47 ± 0.10	20.13 ± 0.07	7.56 ± 0.03	1360.00 ± 20.55	1625.86 ± 122.51
	25 °C	34.50 ± 0.09	25.03 ± 0.10	7.54 ± 0.03	1343.33 ± 30.31	1473.58 ± 130.69

Supplementary material - Table 2. Primers used for qPCR amplification.

Genes (**Accession no.**)	Primer	DNA sequences
SOD (**KX302627**)	Forward	5′- TGC TGA GAG GTG ATT CGG AA -3′
	Reverse	5′- TGT TGT CCC CGA ACT GAT GA -3′
CAT (**OK042347**)	Forward	5′- ATG GTG ACA GGT ATT GAG GC -3′
	Reverse	5′- GAA GTT GCA GGT AGT TGC TG -3′
Caspase-3 (**MT506591**)	Forward	5′- ACG AAA AGG TTC CTG GTT CA -3′
	Reverse	5′- CAG GAT AAT CAT GGG CGA CT -3′
β-actin (**MW387000**)	Forward	5′- GAT AGT GCG AGA CAT CAA GG -3′
	Reverse	5′- CGA TAG TTA TCA CAG ACC CG -3′
For *in situ* hybridization		
SOD (**KX302627**)	Forward	5′- AGC AGT TTG TGT GCT GAG AG -3′
	Reverse	5′- CCT GTC TTC AGG CTT TCT TC -3′

Supplementary material - Table 3. Three-way ANOVA summary of the effects of temperature, pH and time on levels of malondialdehyde (MDA), hydrogen peroxide (H_2O_2) in hemolymph and mRNA expressions of superoxide dismutase (SOD), catalase (CAT) and caspase-3 in hepatopancreas of *H. discus hannai*. The symbol "*" indicates a significant difference. (pH: 8.1, 7.5; temperature: 15, 20, 25 ℃; Time: 0, 1, 3, 5 days; MS: mean square; F: f statistic; P: p-value)

		**pH**	**Time**	**Temp.**	**pH *Time**	**Time *Temp.**	**pH*Temp.**	**pH*Time *Temp.**
**MDA**	MS	98.219	92.015	38.380	27.482	2.0807	4.326	3.345
	F	136.437	127.820	53.314	38.176	2.890	6.009	4.642
	P	<0.001*	<0.001*	<0.001*	<0.001*	0.034*	0.005*	0.004*
**H_2O_2**	MS	11593.215	11683.092	19162.055	762.294	1020.271	899.971	242.251
	F	50.367	50.757	83.250	3.312	4.433	3.910	1.052
	P	<0.001*	<0.001*	<0.001*	0.043*	0.025*	0.003*	0.388
**SOD**	MS	239.816	1254.625	1647.694	15.669	244.670	198.409	4.562
	F	28.004	146.505	192.405	1.830	28.571	23.169	0.533
	P	<0.001*	<0.001*	<0.001*	0.174	<0.001*	<0.001*	0.712
**CAT**	MS	389.147	1244.035	1379.665	13.868	212.769	51.719	18.939
	F	103.230	330.007	365.985	3.679	56.442	13.720	5.024
	P	<0.001*	<0.001*	<0.001*	0.034*	<0.001*	<0.001*	0.002*
**Caspase-3**	MS	1.333	4.112	1.930	0.810	0.569	0.019	0.046
	F	101.195	312.076	146.501	61.482	43.170	1.405	3.493
	P	<0.001*	<0.001*	<0.001*	<0.001*	<0.001*	0.257	0.015*
